# Sequencing bilateral robot-assisted arm therapy and constraint-induced therapy improves reach to press and trunk kinematics in patients with stroke

**DOI:** 10.1186/s12984-016-0138-5

**Published:** 2016-03-22

**Authors:** Yu-wei Hsieh, Rong-jiuan Liing, Keh-chung Lin, Ching-yi Wu, Tsan-hon Liou, Jui-chi Lin, Jen-wen Hung

**Affiliations:** Department of Occupational Therapy and Graduate Institute of Behavioral Sciences, College of Medicine, and Healthy Aging Research Center, Chang Gung University, 259 Wenhua 1st Rd, Taoyuan, Taiwan; School of Occupational Therapy, College of Medicine, National Taiwan University, Taipei, Taiwan; Division of Occupational Therapy, Department of Physical Medicine and Rehabilitation, National Taiwan University Hospital, Taipei, Taiwan; Department of Physical Medicine and Rehabilitation, Shuang Ho Hospital, Taipei Medical University, Taipei, Taiwan; Department of Rehabilitation, Chang Gung Memorial Hospital-Kaohsiung Medical Center, Kaohsiung, Taiwan

**Keywords:** Stroke, Sequential combination therapy, Robotic rehabilitation, Constraint-induced, Upper extremity

## Abstract

**Background:**

The combination of robot-assisted therapy (RT) and a modified form of constraint-induced therapy (mCIT) shows promise for improving motor function of patients with stroke. However, whether the changes of motor control strategies are concomitant with the improvements in motor function after combination of RT and mCIT (RT + mCIT) is unclear. This study investigated the effects of the sequential combination of RT + mCIT compared with RT alone on the strategies of motor control measured by kinematic analysis and on motor function and daily performance measured by clinical scales.

**Methods:**

The study enrolled 34 patients with chronic stroke. The data were derived from part of a single-blinded randomized controlled trial. Participants in the RT + mCIT and RT groups received 20 therapy sessions (90 to 105 min/day, 5 days for 4 weeks). Patients in the RT + mCIT group received 10 RT sessions for first 2 weeks and 10 mCIT sessions for the next 2 weeks. The Bi-Manu-Track was used in RT sessions to provide bilateral practice of wrist and forearm movements. The primary outcome was kinematic variables in a task of reaching to press a desk bell. Secondary outcomes included scores on the Wolf Motor Function Test, Functional Independence Measure, and Nottingham Extended Activities of Daily Living. All outcome measures were administered before and after intervention.

**Results:**

RT + mCIT and RT demonstrated different benefits on motor control strategies. RT + mCIT uniquely improved motor control strategies by reducing shoulder abduction, increasing elbow extension, and decreasing trunk compensatory movement during the reaching task. Motor function and quality of the affected limb was improved, and patients achieved greater independence in instrumental activities of daily living. Force generation at movement initiation was improved in the patients who received RT.

**Conclusion:**

A combination of RT and mCIT could be an effective approach to improve stroke rehabilitation outcomes, achieving better motor control strategies, motor function, and functional independence of instrumental activities of daily living.

**Trial registration:**

ClinicalTrials.gov. NCT01727648

## Background

Stroke remains a leading cause of permanent motor disability worldwide [1]. Persistent impairment of the upper extremity (UE) occurs in up to two-thirds of patients after stroke [2]. UE paresis can lead to deficits in motor control [3], motor dysfunction [4], and participation in activities of daily living (ADL) [5]. Developing and providing effective therapeutic techniques to improve UE motor control and recovery is crucial.

Robot-assisted therapy (RT) is an emerging intervention approach that provides high-intensity, high-repetition, and task-specific training to enhance motor learning and control in patients with stroke [6, 7]. Systemic reviews have indicated that RT improves UE muscle strength and motor function of patients with moderate to severe motor impairment after stroke [8, 9]. A recent review suggested that the assessment of movement kinematics should be included in RT studies to identify modulation in motor control strategies [10]. Previous studies found that RT can improve motor control strategies in patients with stroke, including greater movement efficacy [11–13], better movement smoothness of the affected UE [13], and more use of the preplanned control strategy [13]. However, no consistent findings on patients’ participation in ADL were observed after RT [8, 14–17]. How to optimize or transfer the treatment benefits of RT on motor function and motor control strategies into participation in ADL warrants further investigation. An approach using RT monotherapy may not optimally address this need.

Constraint-induced therapy (CIT), one most investigated approaches to rehabilitation, was developed to overcome the learned nonuse phenomenon and enhance functional use of the affected arm after stroke [18, 19]. Treatment components of CIT include repetitive and intensive task practice, behavioral shaping techniques, restraint of the unaffected UE, and transfer package [20, 21]. Modified and distributed CIT, which are not as intensive as the original CIT, have been developed and validated [20, 22, 23]. The benefits of the original CIT and its modified versions have been well demonstrated to improve motor function, arm-hand activities, and daily performance of patients with stroke [19, 24, 25].

Therapies that combine RT with other rehabilitation approaches have been developed to optimize the treatment effects of RT [26–29]. The combination of RT and conventional therapy led to significant gains in arm function of patients, but different combination sequences showed benefits in different outcomes [27]. In addition, RT combined with repetitive task practice was effective in enhancing hand function and stroke recovery of patients [28]. To the best of our knowledge, only one study has investigated the treatment effects of sequencing the combination of RT and a modified form of CIT (mCIT) in patients with stroke [29]. The results indicated that the sequential combination of RT and mCIT led to better motor and functional ability measured by clinical scales compared with RT alone or conventional rehabilitation [29]. However, whether the changes in motor control strategies are responsible for the improvements in motor function after the sequential combination therapy remains unclear.

Kinematic analysis has been recommended as a sound measure to provide objective and sensitive evaluations on spatial and temporal characteristics of UE movements [8]. More importantly, kinematics can capture motor control strategies that cannot be detected by clinical scales [30]. Thus, kinematic analysis enables us to understand whether the behavioral improvement is due to a true change in the end point control and joint motion or is a result of compensation. Kinematic measures, along with clinical assessments, can better clarify the motor control strategies underlying the motor improvements of stroke patients [31, 32].

This study investigated the effects of the sequential combination of RT and mCIT (RT + mCIT), compared with RT alone, focusing on motor control strategies measured by kinematic analysis and on motor and ADL functions using clinical measures. We hypothesized that (1) RT + mCIT would lead to different benefits on the motor control strategies compared with and RT alone and that (2) RT + mCIT would contribute to better performances in ADL than RT alone.

## Methods

### Design

The data of the current study were derived from part of a single-blinded, randomized controlled trial designed to comprehensively examine the effects of RT alone and the combination of RT and mCIT in patients with stroke. According to previous findings, the estimated sample size required at least 15 patients in each intervention group [29]. The Institutional Review Board of the National Taiwan University Hospital (IRB#201112104RIB) and Chang Gung Memorial Hospital (IRB#99-0832B) approved the study, and all participants signed an informed consent.

### Participants

The study included 34 patients with stroke. The inclusion criteria were chronic unilateral stroke (>6 months poststroke), an initial Fugl-Meyer Assessment (FMA) score between 20 and 50 [33], able to perform ≥10° of wrist extension with extension of at least two fingers >0° and <10° and with thumb abduction ≥10° [34], without excessive spasticity in any of the UE joint (modified Ashworth scale ≤3), without UE fracture within 3 months or painful arthritis, and Mini-Mental State Examination score ≥22.

### Randomization

When a new eligible participant was registered, the participant was stratified into four strata based on the lesion side and the motor impairment level (the cutoff point was 35 in the initial core of the FMA) [33]. An investigator who was not involved in the evaluation and treatment managed the randomization procedure by using a random-number table. Sequentially numbered, sealed, and opaque envelopes containing the group sheets were prepared before the study began.

### Interventions

Participants in RT + mCIT and RT groups received a similar amount of therapy time (an average of 90 to 105 min/day, 5 days for 4 consecutive weeks).

#### RT group

Participants in the RT group used the Bi-Manu-Track (Reha-Stim Co., Berlin, Germany) to perform movements of forearm pronation-supination and wrist flexion-extension. There were three computer-controlled modes. In the passive–passive mode, the device passively moves both arms. In the active–passive mode, the unaffected arm actively drives the affected arm to move passively. In the active–active mode, the affected arm has to overcome the initial resistance to allow the arm movements [35]. Before RT, participants had 5 to 10 min of mobilization as a warm-up. Then, the RT protocol included 600 to 800 repetitions of the passive–passive and active–passive modes for 15 to 20 min and 150 to 200 repetitions of the active-active mode for 3 to 5 min [36]. While the patient’s affected arm can actively perform the movements or as the patient improved, the active-passive mode was adjusted to the affected arm actively driving the unaffected arm to encourage more active movements of the affected arm. After RT training protocol, participants practiced functional-based activities for 15 to 20 min, such as picking up coins, opening a jar, turning pages of newspaper, carrying objects, and twisting a towel, which were selected by the patient and the therapist.

#### RT + mCIT group

For the first 2 weeks, participants in RT + mCIT group received RT, using the same treatment principles as those in the RT group. RT was followed by 2 weeks of a form of mCIT with reduced training and restraint time compared with the original CIT. Treatment components included repetitive training of the affected UE in functional tasks with behavior shaping [29, 37, 38]. A mitt was used to restrict the unaffected hand for 6 h each day [37, 38]. Some strategies of transfer package applied to facilitate the use of the affected UE included behavioral contract, home diary, and problem solving mentoring [39]. The functional tasks included, for example, reaching to move a cup, picking up a utensil to get food, flipping pages of magazines, pouring water, wiping a table, and using a cellphone. The shaping techniques involved individualized task selection, graded task difficulty, verbal feedback, prompting, physical assistance with movements, and modeling. The level of challenge was adapted according to the patient’s ability and progress.

### Outcome measures

The outcome measures were administered before and after intervention by the same blinded assessor. The primary outcomes were kinematic parameters. For the secondary outcomes, the Wolf Motor Function Test (WMFT), Functional Independence Measure (FIM), and Nottingham Extended Activities of Daily Living (NEADL) were used to measure the activity and participation levels [40].

#### Kinematic evaluation

A task of reaching to press a desk bell was used to obtain reaching kinematic performance. The bell was placed along the participant’s midsagittal plane at a distance measured from the media border of axilla to the distal wrist crease. The participant sat on a chair in front of a table with the seat height adjusted to the lower leg’s length. The initial position of the hand was on the table edge with elbow flexed at 90°. The participant was asked to use the index finger of the affected arm to press the bell as quickly as possible [41].

A total of 13 markers were placed on the affected side to model arm and trunk movements, including the spinal processes of the 7th cervical vertebra (C7) and 4th thoracic vertebra (T4), midsternum, bilateral clavicular heads and acromions, the anterior aspect of the upper arm midway between the acromion and the lateral epicondyle, lateral epicondyle, styloid processes of ulna and radius, thumb nail, and the index nail. The marker positions in 3-dimensional space were measured with a sampling rate of 120 Hz by a 7-camera motion capture system (VICON MX, Oxford Metrics Inc, Oxford, UK) and low-pass filtered at 5 Hz using a second-order Butterworth filter. LabVIEW software (National Instruments Inc, Austin, TX) was used to process the kinematic data. Movement onset was defined as the time at which the tangential velocity rose above 5 % of the peak tangential velocity of the markers on the index nail or sternum, and movement offset was defined as the time at which the tangential velocity fell and remained below 5 % of the peak tangential velocity [31].

#### Clinical assessment

We used the WMFT to quantify UE motor function of participants through 15 function-based tasks. WMFT tasks 1 to 6 are timed joint-segment movements, and tasks 7 to 15 are timed integrative functional movements. Participants were scored based on the performance time (WMFT-TIME) and quality of movements (WMFT-Functional Ability Scale [FAS]). Reliability and validity of the WMFT-TIME and WMFT-FAS have been well established [42, 43].

The FIM was used to measure functional independence of basic ADL. It consists six subscales, including self-care, sphincter control, transfer, locomotion, communication, and social ability [44]. The FIM has good inter-rater reliability, construct validity [45, 46], and discriminate validity [47] in patients with stroke.

The NEADL measures instrumental ADL function in patients with stroke [48], which may relate to quality of life after stroke [49]. The NEADL incorporates 22 activities contained in four subscales: mobility, domestic, leisure, and kitchen. The NEADL is a valid measure for evaluating rehabilitation efficacy [50, 51].

### Data reduction for kinematic variables

The kinematic variables used in this study were categorized into three motor control strategies: endpoint coordinate strategy, joint angle coordinate strategy, and trunk compensatory movement [52]. The variables to measure endpoint coordinate strategy include index movement time (Index MT), movement distance (Index Dist), peak velocity (Index PV), and the percentage of movement time at which index peak velocity occurs (Index PPV). These variables were computed according to the marker placed on the index nail. Index MT was defined as the time between the onset and offset of the index movement. The direct distance of the markers placed on the index nail, calculated from index movement onset to offset during the reaching task, was termed Index Dist [53]. The highest instantaneous velocity during the task was defined as Index PV [38, 54]. The percentage of the Index MT at which the index PV occurs represented the Index PPV [55].

**Fig. 1:**
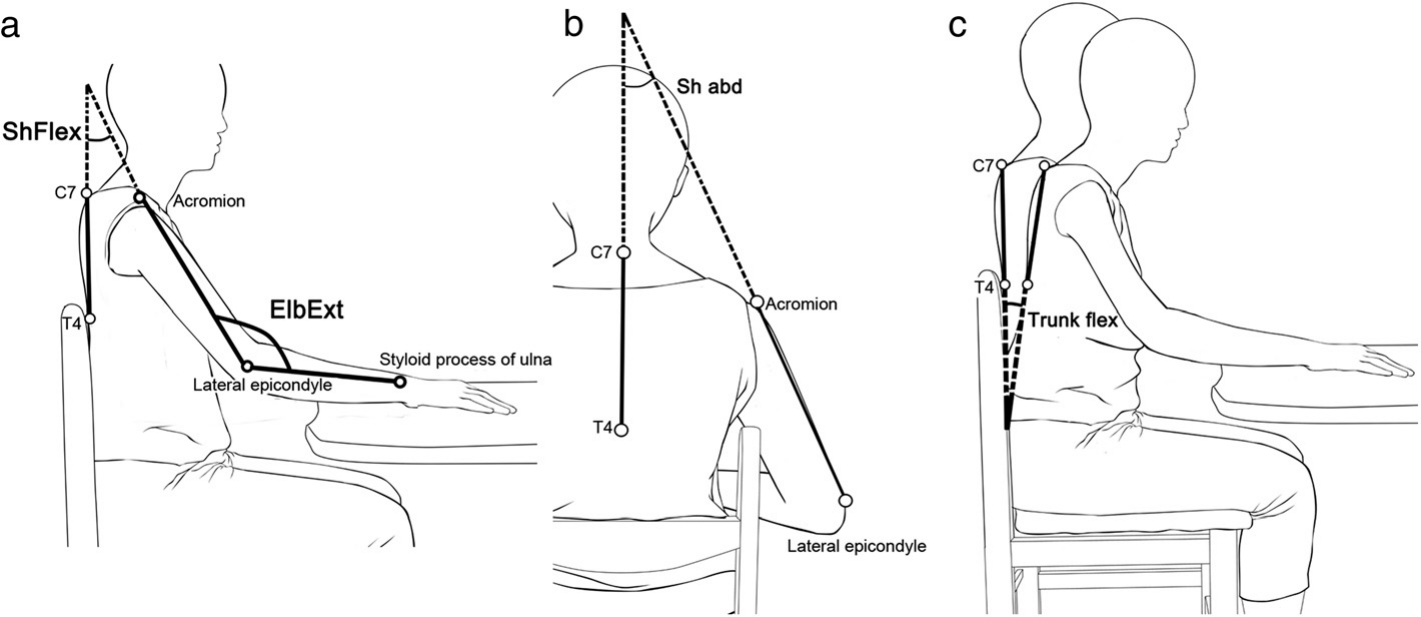
Graphic representation of the angular strategy variables: (**a**) shoulder flexion (ShFlex) in the sagittal plane and elbow extension (ElbExt) in the sagittal plane; (**b**) shoulder abduction (ShAbd) in the frontal plane; and (**c**) trunk flexion in sagittal (TrunkFlex) plane

The shoulder joint angle coordinate strategies can be described by the maximal angle of shoulder flexion (MaxShFlex) and the maximal shoulder abduction (MaxShAbd). The elbow joint angle coordinate strategy can be described by the maximal angle of elbow extension (MaxElbExt). Shoulder flexion was defined as the angle between vectors of the ipsilateral acromion-lateral epicondyle markers and the C7-T4 trunk markers on the sagittal plane (Fig. 1a). Shoulder abduction was calculated as the angle between the vectors of the ipsilateral acromion-lateral epicondyle markers and the C7-T4 trunk markers on the frontal plane (Fig. 1b). The angle between the vector defined by the lateral epicondyle and the styloid process of the ulna and the vector formed by the ipsilateral acromion-lateral epicondyle markers was defined as the elbow angle (Fig. 1a).

Trunk compensatory movement was described by trunk MT (Trunk MT), trunk Dist (Trunk Dist), trunk PV (Trunk PV), and maximal angle of trunk flexion (MaxTrunkFlex) in the sagittal plane. These variables were defined by the marker placed on the sternum. The time between the onset and offset of the trunk movement was defined as Trunk MT. The direct distance of the marker placed on the sternum from trunk movement onset to offset during the reaching task was defined as Trunk Dist. The highest instantaneous velocity during the reaching task was defined as Trunk PV. The angle between the vectors joining the C7-T4 markers at the trunk movement onset and offset was defined as the trunk angle (Fig. 1c).

### Statistical analysis

To compare treatment effect between the two groups, we used analysis of covariance (ANCOVA) for each variable. The baseline performance was treated as the covariate for controlling pretreatment differences. An effect size of partial η^2^ was calculated [56] to index the magnitude of group differences in kinematic performance and clinical scales. The level of statistical significance was set at .05.

## Results

A total of 34 patients participated in this study, and each intervention group had 17 participants. No significant differences between the two groups were found in baseline characteristics (Table 1).

**Table 1:** Demographic and Clinical Features of the Participants for the two Groups

Variables	RT + mCIT	RT	*P* Value
	(*n* = 17)	(*n* = 17)	
Age (years)	55.1 ± 9.4	52.6 ± 13.6	.53
Time after stroke (months)	20.2 ± 13.6	24.8 ± 14.4	.35
Gender			.45
Male	11 (64.7)	13 (76.5)	
Female	6 (35.3)	4 (23.5)	
Side of lesion			.72
Right	6 (35.3)	7 (41.2)	
Left	11 (64.7)	10 (58.8)	
Stroke subtype			.08
Ischemic	8 (47.1)	13 (76.5)	
Hemorrhagic	9 (52.9)	4 (23.5)	
Stroke site			.31
Cortical	10 (58.8)	10 (58.8)	
Subcortical	5 (29.4)	7 (41.2)	
Pons	2 (11.8)	0 (0)	
Initial FMA score	31.6 ± 7.5	35.9 ± 9.5	.15

NOTE. Values are mean ± SD or n (%)

*Abbreviations FMA* Fugl-Meyer Assessment, *mCIT* a modified form of constraint-induced therapy, *RT* robot-assisted therapy, *SD* standard deviation

### Kinematic analysis

Table 2 summarizes the descriptive statistics and results of the ANCOVA that tested the effects of RT + mCIT relative to RT on the kinematic variables.

**Table 2:** Descriptive and Inferential Statistics for Kinematic variables

Kinematic variables	Pretreatment		Posttreatment		ANCOVA		
	RT + mCIT	RT	RT + mCIT	RT	*F*	*P*	Partial *η* ^*2*^
	(*n* = 17)	(*n* = 17)	(*n* = 17)	(*n* = 17)			
Endpoint coordinate strategy variables							
Index MT (ms)	2.53 ± 1.22	2.17 ± 1.59	2.32 ± 1.28	1.84 ± 0.99	0.89	.35	0.03
Index Dist (mm)	252.28 ± 54.84	241.68 ± 49.17	264 ± 48.06	242.61 ± 59.74	0.94	.34	0.03
Index PV (mm/ms)	705.7 ± 279.54	724 ± 201.91	700.42 ± 231.40	847.84 ± 224.96	5.63	.02*	0.15
Index PPV (%)	26.7 ± 18.46	22.12 ± 12.93	25.87 ± 13.77	22.34 ± 14.62	0.35	.56	0.01
Angular coordinate strategy variables							
MaxShFlex (degree)	33.22 ± 25.02	27.16 ± 15.84	32.36 ± 19.52	29 ± 18.31	0.01	.91	<0.001
MaxShAbd (degree)	44.98 ± 18.29	40.19 ± 14.39	39.6 ± 16.45	45.4 ± 13.39	6.10	.02*	0.16
MaxElbExt (degree)	87.43 ± 17.98	88.54 ± 17.2	94.01 ± 18.43	84.5 ± 14.73	4.48	.04*	0.13
Trunk compensatory variables							
Trunk MT (ms)	2.66 ± 1.27	2.21 ± 1.63	2.54 ± 1.44	1.86 ± 1.07	1.58	.22	0.05
Trunk Dist (mm)	95.6 ± 51.22	83.12 ± 34.15	82.76 ± 30.09	91.02 ± 30.98	5.23	.03*	0.14
Trunk PV (mm/ms)	129.01 ± 64.14	122.98 ± 41.89	126.48 ± 49.91	147.01 ± 43.96	4.51	.04*	0.13
MaxTrunkFlex (degree)	10.72 ± 6.69	10.38 ± 5.77	9.54 ± 5.27	13.03 ± 5.39	7.13	.01*	0.19

NOTE: Values are mean ± standard deviation. *P < 0.05

*Abbreviations Dist* distance, *MaxElbExt* maximal angle of elbow extension, *MaxShAbd* maximal angle of shoulder abduction, *MaxShFlex* maximal angle of shoulder flexion, *MaxTrunkFlex* maximal angle of trunk flexion, *mCIT* a modified form of constraint-induced therapy, *MT* movement time, *PPV* percentage of movement time where peak velocity occurs, *PV* peak velocity, *RT* robot-assisted therapy

The results for endpoint coordinate strategy variables showed that the Index PV in the RT + mCIT group was significantly smaller than in the RT group (*P* = .02). Nonsignificant and small effects were found for the Index MT, Index Dist, and Index PPV. The RT + mCIT group showed lower movement impulse for movement initiation during the task of reaching to press the desk bell after treatment than the RT group, but the difference in relative time for online error correction was not significant.

The results for joint angle coordinate strategy variables showed that the MaxShAbd in RT + mCIT group was significantly smaller than in the RT group (*P* = .02), whereas the MaxElbExt in the RT + mCIT group was significantly greater than in the RT group (*P* = .04).

For trunk compensatory movement, the results showed that the Trunk Dist, Trunk PV, and MaxTrunkFlex of the RT + mCIT group were significantly less than in the RT group (*P* = .03, *P* = .04, and *P* = .01, respectively). After treatment, the RT + mCIT group used less trunk movement and had lower trunk movement impulse for movement initiation than the RT group. No significant difference between groups was found for Trunk MT. Compared with the RT group, patients in the RT + mCIT group extended their elbow more and recruited less trunk movements to perform the reaching task after treatment. No significant group difference was found in the MaxShFlex.

### Clinical assessment

The intervention effects of RT + mCIT and RT on the WMFT, NEADL, and FIM are presented in Table 3. The RT + mCIT group demonstrated significantly greater improvements on WMFT-FAS and the NEADL total score than the RT group (*P* = .01 and *P* = .02, respectively). The differences in the WMFT-TIME and the FIM total score between the two groups were not significant.

**Table 3:** Descriptive and Inferential Statistics for Clinical Assessments

Outcome	Pretreatment		Posttreatment		ANCOVA		
	RT + mCIT	RT	RT + mCIT	RT			
	(*n* = 17)	(*n* = 17)	(*n* = 17)	(*n* = 17)	*F*	*P*	Partial *η* ^*2*^
WMFT-FAS	2.21 ± 0.4	2.74 ± 0.68	2.59 ± 0.46	2.89 ± 0.68	6.78	.01*	0.18
WMFT-TIME	6.28 ± 2.22	6.81 ± 5.22	4.84 ± 1.45	7.19 ± 6.66	2.67	.11	0.08
NEADL	20.15 ± 11.24	32.9 ± 14.22	26.44 ± 11.82	33.89 ± 15.31	6.35	.02*	0.17
FIM	119.47 ± 3.99	116.18 ± 7.08	122 ± 2.78	117.53 ± 7.13	3.61	.07	0.10

NOTE. Values are mean ± standard deviation. *P < 0.05

*Abbreviations FAS* functional ability score, *FIM* Functional Independence Measure, *mCIT* a modified form of constraint-induced therapy, *NEADL* Nottingham Extended Activities of Daily Living, *RT* robot-assisted therapy, *SD* standard deviation, *WMFT* Wolf Motor Function Test

## Discussion

This study is the first to investigate the changes in motor control strategies along with motor function and ADLs after RT + mCIT in patients with stroke. Patients who received RT + mCIT or RT alone demonstrated different benefits on motor control strategies after treatment. Compared with the RT group, the RT + mCIT group used less shoulder abduction, more elbow extension, and less trunk compensation movement, representing restoration of better motor control strategies. In contrast, the RT group showed significantly improved force generation at movement initiation than the RT + mCIT group. The RT + mCIT group achieved greater improvements than the RT group on movement quality of the affected limb (measured by the WMFT-FAS) and independence of instrumental ADL (measured by the NEADL). Better UE motor control strategies may contribute to the improvement of UE movement quality and participation in ADLs after RT + mCIT.

### Benefits of RT + mCIT in kinematics relative to RT

RT + mCIT led to better motor control strategies by reducing shoulder abduction, increasing elbow extension, and decreasing trunk compensatory movement during the reaching task than RT. Excessive shoulder abduction and trunk movement during reaching are common compensatory movements after stroke [57]. Our findings suggest that RT + mCIT may promote normalized movement at the shoulder, elbow, and trunk, which may decrease compensatory movement during the reaching task. The kinematics improvement may be associated with the improvement in endpoint coordinate control after RT, and the improvement became a preparation for subsequent mCIT, which continuously strengthen the joint angle coordinate through massed practice of the affected arm with purposeful functional activities training.

RT provides distal movement training with a constant velocity and a high repetition of passive or active movement, which may also help prevent inappropriate compensatory strategies [27]. Moreover, RT + mCIT induced significant improvement on the maximal angle of elbow extension. Previous studies of RT and CIT monotherapy demonstrated beneficial effects on motor control strategy based on kinematic data. Ellis et al. also found that reaching range of motion (including elbow extension) was improved after the intervention with the ACT^3D^ robotic device in stroke patients [58]. They indicated that increased reaching ability may be attributed to the improvements in shoulder-elbow coordination or joint control after RT. In addition, previous CIT studies found that CIT induced feedforward control strategy (more preprogrammed movement) and better spatiotemporal control of movement resulting in improvements in shorter reaction and movement time, smoother trajectories, and better joint coordination [38, 57, 59, 60]. However, CIT did not significantly improve the angle of elbow extension during reaching movements [57, 59]. Extending the affected elbow when reaching outward is difficult for patients with stroke due to the strong synergistic joint torque coupling of shoulder abduction and elbow flexion [61, 62]. Encouragingly, our results showed that RT + mCIT increased elbow extension and reduced shoulder abduction and trunk compensatory movement during the reaching task. An interaction between RT and mCIT likely led to synergistic effects on joint angle coordinate strategies and trunk compensatory movement.

### Benefits of RT in kinematics relative to RT + mCIT

Our results showed that RT led to gains on endpoint coordinate strategy, as reflected by the Index PV variable during the reaching task, suggesting that RT generated more force at movement initiation than RT + mCIT. The results agree with previous studies that showed distal upper-limb RT had significant benefits on increasing muscle strength of patients [35, 63]. These findings suggest that RT is crucial for enhancing force generation, which may lead to the superior effect on endpoint coordinate strategy of RT group in this study.

### Benefits of RT + mCIT in motor function and ADL as measured by clinical scales relative to RT

The RT + mCIT group improved more on motor function measured by the WMFT-FAS than the RT group. This finding was similar to the results of a previous study that found greater motor improvement after RT in sequential combination with mCIT than a control therapy [29]. The RT + mCIT group had greater improvement than the RT group in instrumental ADL function. The mean change score of the NEADL was 6.29 points, which exceeded the minimal clinically important difference (6.1 points) [64]. The possible explanation is that the 2-week mCIT program the patients received focused on the repeated practice of functional tasks, which facilitated transferring the gains in motor performance to instrumental ADL. Further, the improvement of instrumental ADL after RT + mCIT may also transfer to the functional use of the affected arm in executing daily activities that can be detected by the Motor Activity Log [65]. Further research to incorporate the use of the Motor Activity Log to more comprehensively measure daily function of patients is suggested.

No significant group differences were noted in basic ADL function, possibly because there may be an approaching ceiling effect on the total score of FIM in the current study, which did not leave much room for improvement. Our participants were also in the chronic phase of stroke, a phase in which basic ADL functions tend to be stable. In addition, some strategies of CIT transfer package, such as home skill assignment and home practice, were not applied in this study. Transfer package is a set of strategies to facilitate the transfer of CIT treatment gains to real-life activities [39]. Studies showed that larger improvements with long-term effects occurred in the CIT groups that received transfer package compared with groups that did not [21, 66]. Further investigation that includes whole strategies of transfer package [39] may demonstrate greater differences in daily performance between groups.

### Study limitations

One potential confounding factor was that the baseline NEADL score in the RT + mCIT group was significantly lower than in the RT group. Although the improved score of the RT + mCIT group on the NEADL was significantly higher than the RT group, caution is needed in interpreting the results.

Secondly, the lack of a follow-up assessment might limit the understanding of potential long-term effects of combined rehabilitation therapy. Future research should examine the retention effects of therapeutic gains on motor control mechanisms in patients with stroke after combined interventions.

Third, further research with a larger sample size is suggested to apply principal component analysis to help identify the critical components that can represent various kinematic variables and to avoid multiple comparisons of kinematic variables. Use of the ISB standards [67] is suggested for defining joint coordinate systems when reporting kinematic data, which can enhance communication among clinicians and researchers.

In addition, the robot-assisted device used in this study provided training of wrist and forearm movements only. Further studies to investigate the effects of RT focus on the proximal versus distal portion of the UE on motor control strategies are suggested.

## Conclusion

This study demonstrated that patients who received RT + mCIT and RT alone had different aspects of benefits on motor control strategies. RT + mCIT uniquely improved motor control strategies of the affected limb and enhanced independence in instrumental ADL. RT improved force generation at movement initiation. A combination of RT and mCIT contributes to improve motor control strategies, motor function, and functional independence of instrumental ADL for patients with stroke.

## Abbreviations

ADL: activities of daily living
Dist: movement distance
FAS: functional ability score
FIM: Functional Independence Measure
FMA: Fugl-Meyer Assessment
MaxElbExt: the maximal angle of elbow extension
MaxShAbd: the maximal shoulder abduction in the frontal plane
MaxShFlex: the maximal angle of shoulder flexion in the sagittal plane
mCIT: a modified form of constraint-induced therapy
MT: movement time
NEADL: Nottingham Extended Activities of Daily Living
PPV: percentage of movement time at which peak velocity occurs
PV: peak velocity
RT: robot-assisted therapy
RT + mCIT: sequential combination of robot-assisted therapy and a modified form of constraint-induced therapy
UE: upper extremity
WMFT: Wolf Motor Function Test
